# Healthcare workforce mobility and organisational turnover in Sweden, 2014–2024: a multi-method analysis across occupations and sectors

**DOI:** 10.1186/s12913-026-15120-x

**Published:** 2026-07-15

**Authors:** Hanne Berthelsen, Linda Corin, Constanze Leineweber, Sara Stjernlöf, Tuija Muhonen, Hugo Westerlund

**Affiliations:** 1 Centre for Work Life and Evaluation Studies (CTA) & Section 4, Faculty of Odontology, Malmö University, Nordenskiöldsgatan 1, Malmö, 205 06 Sweden; 2 Institute of Stress Medicine, Region Västra Götaland, Carl Skottsbergs gata 22B, Göteborg, 413 19 Sweden; 3 Department of Psychology, Stockholm University, Stockholm, 106 91 Sweden; 4 Centre for Work Life and Evaluation Studies (CTA) & Department of School Development and Leadership, Faculty of Education and Society, Malmö University, Malmö, 205 06 Sweden

**Keywords:** Healthcare, Public health, Human resources management, Sectoral mobility, Exit patterns, Actual turnover

## Abstract

**Background:**

Labour shortages and high staff turnover pose major challenges for healthcare systems, yet most existing research relies on cross-sectional data, focuses on single occupations, and examines intention to leave rather than actual mobility. To address these gaps, this two-part study investigates healthcare staff mobility patterns across public and private employers and occupational groups in Sweden over the past decade (labour market mobility perspective), while also examining types of organisational turnover and the exit destinations of staff who voluntarily left their positions (an organisational turnover perspective).

**Methods:**

We employed a quantitative multi-method design covering four occupational groups (medical doctors, registered nurses, assistant nurses, and other licensed healthcare occupations). Consistent with the two-part design, the study combines two complementary components: (i) a national cohort dataset enabling analyses of occupational mobility and transitions between employers (Study A; labour market perspective), and (ii) register-based turnover data combined with organisational exit survey data from a large regional public healthcare provider (Study B; organisational perspective). Together, these two substudies capture mobility at both societal and organisational levels.

**Results:**

Study A showed that healthcare staff mobility remained relatively stable over the past decade, but patterns varied notably by occupation. Most transitions occurred between public and private employment, with private sector employees showing the highest mobility. Nursing staff moved mainly between different public employments, in contrast to the other two occupational groups. Relatively few employees changed occupations, but those who did mostly switched to non-healthcare occupations. Study B revealed that external turnover rates (i.e., employees leaving the regional organisation) exceeded internal rates (i.e., employees changing jobs within the organisation) across all years and occupations; registered nurses had the highest average external turnover rate (8%), and an upward trend was observed for assistant nurses. While most had secured new jobs when leaving, many, especially assistant nurses, left without knowing their future employment situation.

**Conclusion:**

Healthcare staff mobility patterns in Sweden have remained relatively stable from 2014 to 2024, yet distinct patterns across various occupational groups reveal structural vulnerabilities. By integrating labour market and organisational perspectives, this study shows that mobility is not uniform but varies across occupations, sectors, and types of exit pathways.

## Background

The prevailing labour shortages and high staff turnover in healthcare have consequences for patients, staff, and society, and are, as such, significant public health problems [1]. By 2030, the World Health Organisation estimates a shortfall of 10 million healthcare workers [2]. The healthcare workforce faced significant turnover related to the pandemic in the US, as in many other countries [3]. In most European countries, registered nurses (RNs), assistant nurses (AN) and other healthcare professionals are now among the occupations with the most critical shortages [4–6]. Healthcare labour shortages have led to an increased international migration of healthcare workers, resulting in severe consequences for source countries and dependency on foreign-trained staff in recipient countries [2, 4, 7]. This competitive environment manifests not only internationally but also within individual countries. Around two-thirds of the Swedish municipalities reported in 2022 shortages of licensed healthcare staff (mainly RNs, ANs and physiotherapists), and the majority of regional employers reported a lack of midwives, specialised nurses and physicians, psychologists, dentists and dental hygienists [8, 9]. Despite the pandemic being over, the severe healthcare staffing problems persist [10].

A certain level of labour mobility is beneficial and can, for example, enhance professional development and improve the overall efficiency of healthcare systems. However, when demand for skilled healthcare professionals is high, competition can intensify mobility to an excessive extent. There are indications that high staff turnover has negative implications for care quality [11, 12] and is associated with considerable monetary costs for organisations, as well as indirect costs such as loss of competence [13, 14]. It may lead to a vicious spiral of increased workload and stress among the remaining employees, thereby exacerbating the original problem. In Sweden, the situation in the healthcare sector is so critical that the government has launched a national strategy to address the challenges [15]. Further, the Association of Local Authorities and Regions states that the recruitment and retention of skilled professionals in the welfare sector is one of the most pressing issues for the coming decade [16].

Most turnover literature comprises cross-sectional studies on voluntary turnover using turnover intentions (i.e., the desire or intention to change job) as a proxy for actual turnover [17]. Although many studies have demonstrated a link between individuals’ intentions and actual turnover, the generalisation of turnover intentions to situations involving actual turnover remains questionable due to the difference between intent and action [18–20]. Intention to leave is typically more prevalent than the actual behaviour of leaving (e.g [21–23]., though with some exceptions [e.g. 24]. Previous research suggests that, at the organisational level, turnover intentions and actual turnover are distinct constructs predicted by different sets of variables [25]. Yet empirical studies on actual turnover in the public sector are scarce [26], and most evidence originates from the United States and Canada. Therefore, further studies in European contexts, grounded in actual turnover data, are needed.

Within the healthcare sector, research on turnover has primarily focused on a single occupation, despite healthcare organisations comprising mixed, interdependent occupational groups. The most studied occupation is that of RNs, while studies on other occupations remain limited. However, a meta-review of systematic reviews of nurse turnover studies indicated a need for further research, even concerning RNs [27]. This highlights the need for studies employing more robust research designs that analyse actual turnover rates across healthcare occupations.

The Swedish healthcare system is decentralised, with 21 regions and 290 municipalities having primary responsibility for financing, organisation, and service delivery. The system is mainly funded through taxes and aims to provide equitable access to care for all residents [28]. The regions are responsible for hospitals and specialised healthcare and employ the majority of physicians, registered nurses, and assistant nurses. In contrast, municipalities are responsible for the care of people with disabilities and the elderly, as well as school health services, primarily employing nursing staff and relatively few physicians [29]. It should also be noted, that while most Swedish healthcare is publicly funded, a significant share of this publicly funded care is provided by private providers. Mobility between public and private sectors is therefore primarily an organisational employment issue rather than a threat to publicly funded care.

This two-part study investigates healthcare staff mobility in Sweden across public and private employers, and across occupations over the past decade (Study A: labour market mobility perspective), as well as the different kinds of turnover and exit destinations of healthcare staff who have voluntarily left their jobs (Study B: organisational turnover perspective). Both studies cover the same time period and occupational groups and provide complementary insights into healthcare staff mobility. This approach responds to Burr’s call for the “simple but necessary” mapping of basic trends before advanced analyses, and for combining research data with monitoring data to achieve deeper analytical insights [30].

### Brief introduction and research questions for Study A

The nature of turnover in the healthcare and welfare sector is complex and context-specific [31]. While the decision to leave is made by the individual, actual turnover behaviour is significantly affected by broader cultural influences [32]. Recent research from Sweden indicates that exit among nurses is not merely a response to dissatisfaction but reflects a broader cultural shift in how work, loyalty, and professional identity are understood under conditions of marketisation [33]. Against this backdrop, a recent Swedish report on welfare-sector staff statistics shows that significant gaps remain in understanding the patterns and drivers of employee mobility [34]. Current analyses tend to focus exclusively on publicly employed staff, neglecting critical insights into the flow of personnel between public and private employers [34]. This limitation prevents a comprehensive understanding of how this kind of mobility shapes the composition and stability of the workforce.

In 2021, more than 13,000 RNs in Sweden were not employed in their professional roles, resulting in a loss of skills corresponding to an estimated cost to society of just over SEK 5.5 billion [15]. From an individual perspective, pursuing a career may also involve changing occupation. For example, an AN could return to the education system to become an RN, benefiting the healthcare sector. In contrast, pursuing a career in a non-healthcare field would exacerbate the skills shortage. In other words, investigating the extent and nature of healthcare staff mobility across occupations is highly relevant. Against this backdrop, Study A constitutes the labour market perspective of this two-part study and focuses on broader mobility patterns across sectors and occupations over time.

Study A aims to identify and describe trends in healthcare staff mobility patterns over one decade, focusing on medical doctors (MDs), RNs, ANs, and other licensed healthcare occupations (OCs) in Sweden^1^. The specific research questions are:

RQ A.1 What is the extent of healthcare staff mobility across employers in public, private and other *sectors* over the last decade?

RQ A.2 What is the extent of healthcare staff mobility across *occupations* over the last decade?

### Brief introduction and research questions for Study B

Research on turnover is characterised by a lack of conceptual clarity and inconsistency in measurement. Reviews of research on actual turnover rarely make clear distinctions between voluntary and involuntary turnover, or between leaving the unit, the organisation, and the profession [31]. From an organisational perspective, distinguishing between internal and external mobility is particularly critical. Employees who leave for external opportunities may be driven by dissatisfaction with their current roles, better career prospects elsewhere, or a desire for professional advancement. Conversely, according to Bidwell and Mollick [35], those moving to new roles within the same organisation may be motivated by the potential for skill development, career progression, or a better job fit. Failing to distinguish between these forms of mobility risks obscuring whether turnover reflects organisational loss or internal reallocation of competence.

Moreover, turnover is not a uniform outcome but a set of qualitatively different trajectories. Hom et al. underscore the importance of considering employees’ post-departure trajectories [21], noting that the underlying motivations for voluntary turnover vary considerably depending on whether individuals transition to a new employer, assume stay-at-home parenting responsibilities, pursue further education, shift career paths, or retire early. Therefore, a generalised approach to turnover that fails to account for these distinctions may risk leading to ineffective or misdirected retention strategies.

Study B of this two-part study adopts an organisational perspective and aims to investigate voluntary turnover by separating internal and external mobility and mapping employees’ exit destinations.

The research questions are:

RQ B.1 How has voluntary internal and external turnover developed over the last decade for MDs, RNs and ANs?

RQ B.2 What are the exit destinations of healthcare staff (ANs, RNs, MDs, and OCs) who voluntarily left employment within the regional healthcare organisation during 2020–2024?

## Materials and methods

This study employs a multi-method quantitative design comprising two complementary studies (Study A and Study B). Together, they capture healthcare staff mobility from both societal (labour market) and organisational perspectives.

Study A (the labour market perspective) draws on a longitudinal national survey (SLOSH) tracking individuals enabling analyses of movements between occupations and between public and private employment over the past decade. Study B (the organisational perspective) is based on organisational data from a large public healthcare provider, combining HR register data with exit survey data to enable a more detailed examination of turnover patterns and exit destinations within a specific organisational context. Taken together, this two-part design allows for an integrated analysis of mobility, combining broad population-level trends with in-depth organisational insights.

### Material and methods for Study A)

#### Study design

Study A is based on data from SLOSH, the Swedish Longitudinal Occupational Survey of Health [36]. It is an ongoing panel study conducted by the Stress Research Institute at Stockholm University in Sweden, with data collection every second year since 2006, and yearly data collection since 2022 (www.slosh.se). Data is collected through surveys in which participants self-report various aspects of their work and health. The SLOSH sample comprises respondents from the Swedish Work Environment Survey (SWES), who are in turn randomly selected from the Labour Force Survey (LFS), conducted every second year by Statistics Sweden. Consequently, the SWES is based on a nationally representative sample of Sweden’s working population. Today, all eligible SWES participants from 2003 to 2021 are invited to respond to SLOSH questionnaires.

#### Sample

In the current study, we used data from six waves: 2014, 2016, 2018, 2020, 2022, and 2024. Over the study period, the response rate ranged from 52% in 2014 to 45% in 2024. Following our aim, we chose four different occupational groups, namely MDs (SSYK 221), RNs (SSYK 222 and 223), ANs (SSYK 532), and OCs, including psychologists and psychotherapists (SSYK 224), dentists (SSYK 226), chiropractors, occupational therapists and physiotherapists (SSYK 227), other healthcare specialists: Pharmacist, Audiologist, Dietitian, Speech Therapist, Optometrist (SSYK 228), and Dental Hygienists (SSYK 325).

We created five separate samples for pairwise comparisons using respondents who answered the survey in two subsequent biennial waves: 2014 to 2016 (sample 1, *n* = 1294), 2016 to 2018 (sample 2, *n* = 1114), 2018 to 2020 (sample 3, *n* = 885), 2020 to 2022 (sample 4, *n* = 607) and 2022 to 2024 (sample 5, *n* = 884). Individuals had to meet the following criteria to be included in a sample: respondents in paid work in both waves, and aged 20 to 65 at baseline for each sample. A single individual could be included in multiple, or even all, samples. In total, 224 individuals aged 65 or younger responded in all six waves.

#### Measurements

Based on self-reported information, occupation was coded according to the Swedish Standard Classification of Occupations (SSYK 2012). No information was available for 2024. Consistent with the labour market focus of Study A, mobility was operationalised in two ways: (i) sector mobility, operationalised as working in a different sector (public, private, or other) at follow-up, and (ii) occupational mobility, defined as having a different occupation at the subsequent measurement. No information was available to reliably distinguish between voluntary and involuntary mobility.

A variable ‘employment sector’ (public, private, and other) was created based on self-reported information about the main employer. Employer was measured by nine categories, i.e. ‘private’, ‘association/non-profit organisation’, ‘municipality’, ‘county/region’, ‘state’, ‘farmer’, ‘self-employed with employees’, ‘self-employed without employees’, and ‘other’. The respondents were asked to tick only one alternative, but double ticks were quite common. Consequently, those who ticked only ‘private’ were coded as working in the private sector. Those who ticked ‘municipality’, ‘county’, ‘state’, or any combination of these response options were coded as working in the public sector. Those who selected any of the remaining alternatives, or any combination of them, were coded as working elsewhere. A few persons who ticked both private and public options were coded as missing. Mobility to another occupation was defined as having a different healthcare occupation at follow-up than at baseline or as having an occupational SSYK code not included in the inclusion criteria at follow-up.

Age and gender were derived from registry data. Sex is dichotomous (men/women). Age was based on year of birth and categorised into four age groups: 34 years or younger, 35–44 years, 45–54 years, and 55–65 years. Persons aged 65 and older were excluded from the respective wave.

#### Statistical analyses

We calculated the proportion of public sector employees who, by the next time point, either were (1) still employed in the public sector, (2) had transitioned to a private sector employment, or (3) were working in a different part of the labour market (e.g. an NGO). Corresponding calculations were done for private sector employees. All analyses were stratified by occupation (ANs, RNs, MDs, and other licensed occupations). At baseline, all respondents worked in healthcare; however, when changing employers, this could include moving between healthcare and non-healthcare employers.

For each wave stratified by occupational group, occupational mobility was calculated as the proportion of employees who changed to another of the care professions or to an alternative profession.

To test for differences in retention between occupational groups employed in the public and the private sectors, respectively, we used generalised estimating equations (GEEs). GEE adjusts for the dependence of within-person observations over time by assuming a specific correlation structure between the outcome variable at different time points. Analyses were conducted using a logit link function and binomial distribution to predict the likelihood of staying in the sector. A working exchangeable correlation structure was specified to account for within-subject correlations across repeated measurements, assuming a constant correlation between all observations for the same subject. In addition, the parameters were estimated using the Huber-White sandwich estimator, which is robust to a misspecification of the correlation structure—it adjusts the standard errors to reflect the true variation in the estimates. To increase statistical power, we pooled data across multiple waves and analysed whether occupations differed in their retention rates in the public and private sectors. To test whether mobility differed between sectors, a variable indicating exit from each sector was constructed. Next, GEEs were used to analyse whether the ‘risk’ of exit differed between the two main sectors (public and private). Individuals with another employer were excluded from these analyses. Due to the small number of respondents and partially overlapping sample, it was not possible to statistically test the shift of occupation. All analyses were conducted using SAS 9.4.

### Materials and methods for Study B)

#### Study design

Study B draws on two datasets from a Swedish regional healthcare organisation, encompassing public hospitals and primary care, with more than 50,000 employees: (i) HR register data on staff mobility (2014–2024), and (ii) exit survey data collected from 2020 to 2024. The register data covering voluntary turnover rates from 2014 to 2024 was collected from the organisation’s HR analytics group and used to answer the first research question (RQ B.1). To address the second research question (RQ B.2), organisational exit survey data from 2020 to 2024 were analysed to address the second research question. The survey has been continuously distributed to all employees recorded in the HR system as having terminated their contracts since January 2020, obtaining an average response rate of approximately 60%. Grounded in existing turnover research, the survey examines reasons for leaving across the labour market, organisational, job, and individual levels. Additionally, it includes demographic data and details on post-exit destinations, facilitating a comprehensive understanding of turnover dynamics.

#### Study samples

To include HR register data on only voluntary turnover from 2014 to 2024, turnover resulting from the employer terminating employment was excluded (dismissed, dismissal due to lack of work, dismissal for personal reasons, dismissed by agreement), along with turnover due to fixed-term employment ending, contractual pension, employees on full sickness compensation, and deceased. The register data included internal turnover rates, defined as job changes occurring within the regional healthcare organisation, and external turnover rates, defined as leaving the regional organisation for MDs (SSYK 221), RNs (SSYK 222 and 223), and ANs (SSYK 532). Internal turnover comprised both movements within the same division and movements between divisions. Divisions correspond to administratively defined organisational entities, which in the healthcare sector typically represent hospital-level organisations^2^.

To study exit destinations for staff leaving the regional employer (external turnover), exit surveys from January 2020 to May 2024 were available. The following inclusion criteria were used: (i) voluntary turnover, (ii) leaving the regional employer (external turnover) and (iii) a licensed healthcare occupation, including MDs (SSYK 221), RNs (SSYK 222 and 223), ANs (SSYK 532) and OCs (SSYK 224, 226–228, 325). The final sample comprised responses from 4,990 employees who fulfilled these inclusion criteria.

#### Measurement

Consistent with the organisational focus of Study B, voluntary turnover was assessed through the survey question: “Did you end your employment on your own request?” There were three response options: *“Yes”*,* “No”* and *“I have a fixed-term employment that ends without extension.”* Only respondents who answered *“Yes”* were classified as voluntary leavers. Additionally, cases of turnover due to retirement were excluded.

External turnover was measured using the question: “What will be your main occupation after ending your employment at [current workplace]?” Participants could select from the following response options: “*Other employment within [organisation]*,*” “Employed by another employer*,*” “Self-employed/freelance work*,*” “Studies*,*” “Jobseeker*,*” “Retirement*,*” “Yet to be determined*,*”* and *“Other.”* Individuals who changed to other employment within the organisation were excluded. Respondents replying, “*employed by another employer”* got the following extra response options specifying future employer: *“Different Region”*,* “Municipality”*,* “State”*,* “Private”*,* “NGO”*,*” Cooperative”*,* “Foundation”*,* “Leaving the Swedish labour market”*,* or “Other”.* The response option *“Other”* allowed participants to provide open-ended responses that did not align with the fixed options. These responses were reviewed, and participants were reassigned to existing categories when applicable. Additionally, during the data collection period, new categories were introduced to the questionnaire for frequently recurring open-ended responses that did not fit the predefined alternatives. For example, “*Leaving the Swedish Labour market* was added, encompassing respondents who relocated abroad for work, family, or unspecified reasons.

#### Analyses

Consistent with the organisational focus of Study B, HR register data on monthly turnover rates for RNs, MDs, and ANs, were used to calculate the average annual turnover rate for internal movements (within and between divisions), as well as external turnover, for each occupational group.

To analyse exit destination patterns, frequency tables and cross-tabulations were generated for two exit survey questions: “What will be your main occupation after ending your employment at [current workplace]?” and “In which labour market sector will you be working?” Occupation-specific descriptive analyses were conducted to identify similarities and differences in voluntary turnover patterns across professions. Differences between groups were tested with chi-square tests with Bonferroni corrections. All statistical analyses were performed using IBM SPSS Statistics version 28.

## Results

### Sample characteristics, Study A

Table 1 describes the sample characteristics of Study A. Overall, in all two-year periods, more than 8 out of 10 were women, and around half of the respondents were between 55 and 65 years old (44–52%). More than two-thirds were RNs or ANs across the two-year periods, while MDs were the smallest occupational group. More than 80% worked in the public sector (Table 1).

**Table 1:** Descriptive characteristics of the five samples based on the national SLOSH cohort

	Sample 1* (2014-16) *n* = 1,284	Sample 2 (2016-18) *n* = 1,114	Sample 3 (2018-20) *n* = 885	Sample 4 (2020-22) *n* = 607	Sample 5 (2022-24) *n* = 883
	*n* (%)	*n* (%)	*n* (%)	*n* (%)	*n* (%)
**Sex**					
Female	1135 (87.7)	986 (88.5)	781 (88.3)	524 (86.3)	748 (84.6)
**Age groups**					
1 (34 or younger)	86 (6.65)	56 (5.0)	34 (3.8)	20 (3.3)	39 (4.4)
2 (35–44)	182 (14.1)	165 (14.8)	127 (14.4)	84 (13.8)	162 (18.3)
3 (45–54)	451 (34.9)	354 (31.8)	270 (30.5)	188 (31.0)	260 (29.4)
4 (55–65)	575 (44.4)	539 (48.4)	454 (51.3)	315 (51.9)	423 (47.9)
**Occupation**					
Medical doctor	116 (9.0)	105 (9.4)	89 (10.1)	72 (11.9)	104 (11.8)
Registered nurse	459 (35.5)	385 (34.6)	300 (33.9)	229 (37.7)	333 (37.7)
Assistant nurse	486 (37.6)	411 (36.9)	312 (35.3)	177 (29.2)	242 (27.4)
Other licensed healthcare occupations**	233 (18.0)	213 (19.1)	184 (20.8)	129 (21.3)	205 (23.2)
**Work Sector**					
Public	1096 (85.4)	937 (85.0)	742 (84.3)	499 (83.3)	730 (83.5)
Private	141 (11.0)	131 (11.9)	109 (12.4)	80 (13.4)	118 (13.5)
Other	47 (3.7)	34 (3.1)	29 (3.3)	20 (3.3)	26 (3.0)

* For example, Sample 1 provides baseline data for individuals who answered the questionnaire and were in work in 2014 and 2016, were under the age of 65 in 2014, and worked within one of the occupations under consideration

** Other licensed healthcare staff SSYK12 = 224 (Psychologist/Psychotherapist), 226 (Dentist), 227 (Occupational Therapist & Physiotherapist), 228 (Other healthcare specialists: Pharmacist, Audiologist, Dietitian, Speech Therapist, Optometrist), and 325 (Dental Hygienists)

### Sectoral employment mobility (Study A)

Overall, mobility between sectors was limited, and most employees remained in their initial sector across all occupational groups and time periods (see Table 2 for mobility among the four occupational groups separately, based on 2-year periods). Among those employed in the public sector, OCs had the highest mobility out of the public sector (on average 4,5% across the five samples), while ANs had the lowest (on average 1,5% across the five samples). Among all occupational groups, the majority of those leaving the public sector transitioned to the private sector, while only a few moved to other sectors. In contrast, RNs had the highest mobility out of the private sector (on average 20,5% across the five samples), and for all occupational groups, the transition from the private sector was mainly to public sector employment.

**Table 2:** Proportion of healthcare occupation staff groups who have (1) have stayed employed in the public sector (state, region, municipality), (2) who have left the public sector to a private employer, (3) who work elsewhere, (4) who have stayed in the private sector, (5) who have left their employment in the private sector to a public employer, (6) who work elsewhere. Based on the national SLOSH cohort

	1) Still in the public sector				2) Changed to a private employer				3) Changed to work elsewhere			
	MD	RN	AN	OC	MD	RN	AN	OC	MD	RN	AN	OC
	*n* (%)	*n* (%)	*n* (%)	*n* (%)	*n* (%)	*n* (%)	*n* (%)	*n* (%)	*n* (%)	*n* (%)	*n* (%)	*n* (%)
2014–2016	91 (95.79)	378 (97.17)	431 (98.40)	154 (95.06)	2 (2.11)	10 (2.57)	7 (1.60)	5 (3.09)	2 (2.11)	1 (0.26)	0	3 (1.85)
2016–2018	85 (97.70)	309 (97.17)	355 (97.26)	146 (96.05)	1 (1.15)	8 (2.52)	4 (1.10)	5 (3.29)	1 (1.15)	1 (0.31)	6 (1.64)	1 (0.66)
2018–2020	73 (97.33)	241 (96.40)	274 (97.86)	120 (93.75)	2 (2.67)	8 (3.20)	3 (1.07)	6 (4.69)	0	1 (0.40)	3 (1.07)	2 (1.56)
2020–2022	56 (93.33)	177 (96.20)	151 (96.18)	86 (95.56)	3 (5.00)	7 (3.80)	4 (2.55)	4 (4.44)	1 (1.67)	0	2 (1.27)	0
2022–2024	87 (96.67)	262 (95.97)	204 (96.23)	144 (97.30)	2 (2.22)	8 (2.93)	7 (3.30)	4 (2.70)	1 (1.11)	3 (1.10)	1 (0.47)	0

	**4) Still in the private sector**				**5) Changed to a public employer**				**6) Changed to work elsewhere**			
	**MD**	**RN**	**AN**	**OC**	**MD**	**RN**	**AN**	**OC**	**MD**	**RN**	**AN**	**OC**
	**n (%)**	**n (%)**	**n (%)**	**n (%)**	**n (%)**	**n (%)**	**n (%)**	**n (%)**	**n (%)**	**n (%)**	**n (%)**	**n (%)**
2014–2016	13 (72.22)	38 (76.00)	35 (92.11)	26 (86.67)	3 (16.67)	11 (22.00)	2 (5.26)	2 (6.67)	2 (11.11)	1 (2.00)	1 (2.63)	2 (6.67)
2016–2018	12 (92.31)	42 (84.00)	24 (77.42)	33 (91.67)	0	8 (16.00)	5 (16.13)	0	1 (7.69)	0	2 (6.45)	3 (8.33)
2018–2020	8 (80.00)	30 (83.33)	20 (86.96)	31 (83.78)	0	5 (13.89)	3 (13.04)	5 (13.51)	2 (20.00)	1 (2.78)	0	1 (2.70)
2020–2022	9 (90.00)	21 (70.00)	10 (76.92)	21 (87.50)	1 (10.00)	9 (30.00)	3 (23.08)	2 (8.33)	0	0	0	1 (4.17)
2022–2024	10 (90.91)	38 (84.44)	18 (85.71)	34 (85.00)	1 (9.09)	6 (13.33)	3 (14.29)	5 (12.50)	0	1 (2.22)	0	1 (2.50)

Notes:

1. Medical Doctor (MD), Registered Nurse (RN), Assistant Nurse (AN), Other Licensed Care Occupations (OC)

2. Double responses are handled as changing to work elsewhere

Using GEE to account for repeated measures within individuals, we found no significant differences in public sector retention between occupational groups overall using the conservative Wald statistic (Wald χ² = 7.22, df = 3, *p* < .06). However, the less restrictive pairwise comparisons of least squares means indicated significant differences between specific occupation groups. RNs were significantly less likely to leave employment in the public sector than OCs (β=−0.48, *p* = .02), with an estimated 38% lower odds of exiting. ANs also showed a significantly lower likelihood of leaving the public sector than OCs (β=−0.60, *p* < .01), with an estimated 45% reduction in odds. No other pairwise comparisons were statistically significant (all *p* > .18).

Regarding mobility out of employment in the private sector, no statistical differences were found overall with the Wald statistic (Wald χ² = 5.16, df = 3, *p* < .16). However, RNs were significantly more likely to leave the private sector than OCs (β = 0.52, *p* = .04), with an estimated 52% higher odds of exiting.

Comparing the risk of exit between employment in the public and private sectors across all occupations and samples, we found that the odds of exiting the public sector were significantly lower (β = -0.66, *p* < .0001) than those exiting the private sector.

### Occupational mobility (Study A)

The occupational group that changed jobs most frequently over each of the four 2-year periods with available data was ANs (10.3%), compared to 6.2% of MDs (Table 3). Among those changing to alternative occupations, MDs, RNs and OCs most frequently transitioned into healthcare management or advanced healthcare roles (MDs 38%; RNs 49%; OCs 24%), while two-thirds of the ANs moved into adjacent roles such as personal assistants and care aides (66%). However, 29% in total pursued more divergent paths, including roles in retail, agriculture, rail transport, and beauty therapy. Due to the small numbers in partially dependent samples, it was not possible to formally test whether these differences were statistically significant.

**Table 3:** Proportion of medical doctors (MD), registered nurses (RN), assistant nurses (AN), and other licensed healthcare staff (OC) who changed occupation. Based on the national SLOSH cohort

Period	Changing to another caring occupation*				Changing to an alternative occupation			
	MD	RN	AN	OC	MD	RN	AN	OC
	*n* (%)	*n* (%)	*n* (%)	*n* (%)	*n* (%)	*n* (%)	*n* (%)	*n* (%)
2014–2016	1 (0.86)	4 (0.88)	3 (0.62)	0	6 (5.17)	18 (3.92)	39 (8.02)	17 (7.30)
2016–2018	0	1 (0.26)	2 (0.49)	0	4 (3.81)	27 (7.01)	37 (9.00)	9 (4.23)
2018–2020	0	1 (0.33)	0	0	6 (6.74)	15 (5.00)	33 (10.58)	15 (8.15)
2020–2022	1 (1.39)	0	0	0	5 (6.94)	27 (11.79)	22 (12.43)	9 (6.98)

* I.e. changing to another of the occupations included in the present study (MD, RN, AN, OC)

### Development of internal and external organisational turnover 2014–2024 (Study B)

**Fig. 1:**
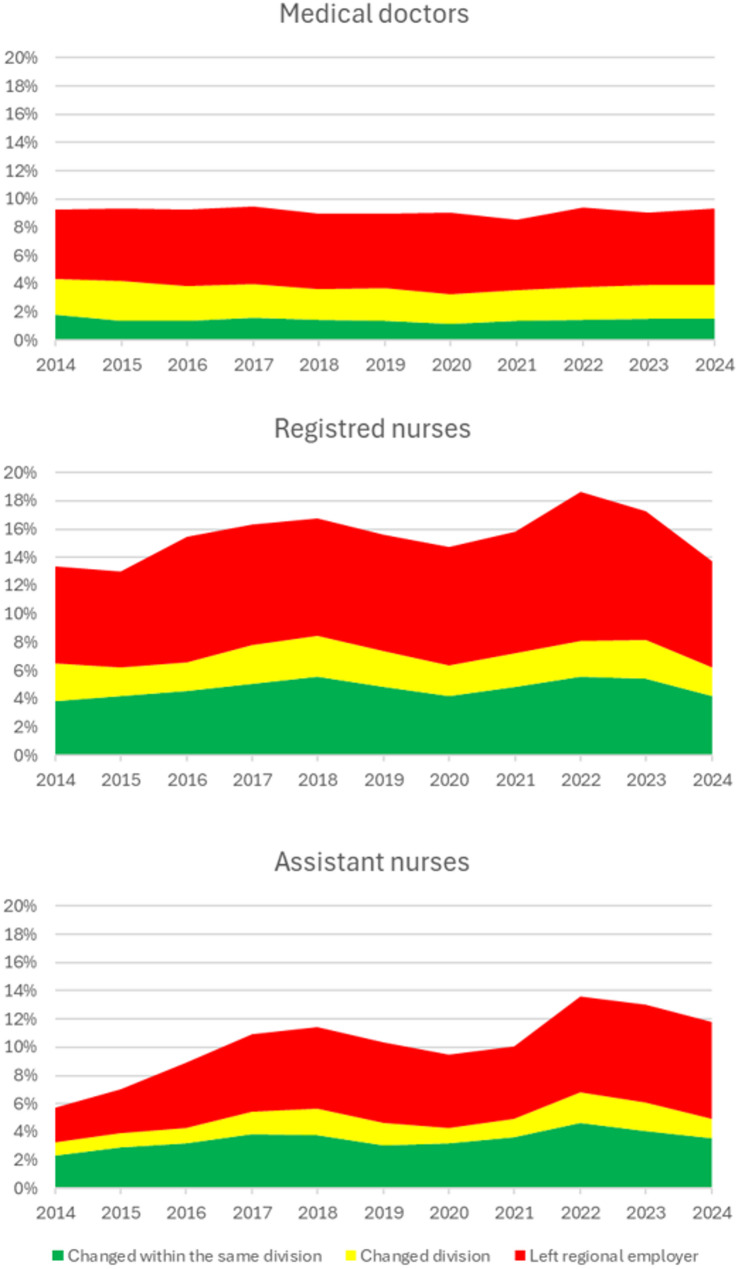
Annual external and internal organisational turnover rates in percentages for medical doctors, registered nurses and assistant nurses during the period 2014–2024. Based on organisational register data

Figure 1 shows the development of internal (within and between divisions) and external (leaving the regional health provider organisation) annual turnover rates for MDs, RNs, and ANs from 2014 to 2024. No corresponding HR-register data were provided for the mixed group of OCs.

Overall, external turnover consistently exceeded internal mobility across all years and occupational groups (MDs, RNs, and ANs), indicating that turnover primarily reflects staff loss rather than internal reallocation. RNs had the highest external turnover rates (average 8%), with a post-pandemic peak in 2022 (11%), followed by a declining trend for 2023-24. The external turnover rates were comparable for MDs and ANs, averaging 5%. However, while stable for MDs, external turnover among ANs increased over time. In terms of internal mobility, RNs and ANs were more likely to move within divisions, whereas MDs more often changed positions between divisions.

### Exit destinations (Study B)

Table 4 presents the exit survey sample of 6,214 licensed healthcare employees, of whom RNs comprised about half of the participants. Most participants were women (80.6%), and the largest age group was 30–39 years (33.5%).

**Table 4:** Descriptive characteristics of the organisational exit survey sample (Study B)

	*n* (%)
**Final Study Population**	6214 (100)
**Sex**	
Male	1024 (16.5)
Female	5009 (80.6)
Other	68 (1.1)
Missing	113 (1.8)
**Age groups**	
2 (< 20)	10 (0.2)
3 (20–29)	1095 (17.6)
4 (30–39)	2080 (33.5)
5 (40–49)	1415 (22.8)
6 (50–59)	1151 (18.5)
7 (60–64)	357 (5.7)
8 (> 64)	98 (1.6)
Missing	8 (0.1)
**Occupation**	
Medical doctor	908 (14.6)
Registered nurse	3109 (50)
Assistant nurse	924 (14.9)
Other licensed healthcare occupations**	1273 (20.5)

** Other licensed healthcare staff SSYK12 = 224 (Psychologist/Psychotherapist), 226 (Dentist), 227 (Occupational Therapist & Physiotherapist), 228 (Other healthcare specialists: Pharmacist, Audiologist, Dietitian, Speech Therapist, Optometrist), and 325 (Dental Hygienists)

Table 5 displays the exit destinations for each occupational group. The pattern of transitions into a new employment, self-employment, studies, unemployment, or undefined plans varied significantly across all occupations (*p* < .01).

### Transitions to new employment outside the organisation (Study B)

As expected, most employees who left the regional organisation had secured employment with a new employer (Table 5, upper part), ranging from 66.2% among ANs to 82.6% among OCs. Clear occupational patterns emerged among those who changed employers (Table 5, lower part). MDs exhibited a significantly different transition pattern from that of other occupational groups. A noTable 47.1% of MDs moved to new employment with another regional employer, while only 2.0% transitioned to municipal positions. In contrast, OCs had the highest percentage, 50.3%, moving to an employer in the private sector.

RNs and ANs exhibited a similar overall pattern, with no significant differences. For both nursing occupations, a substantial percentage shifted to municipal employment (38.4% for RNs and 42.9% for ANs), whereas switching to other regional employers was less common (17.8% for RNs and 12.5% for ANs).

### Alternative career paths (Study B)

As could be expected, MDs had the highest prevalence of transitioning to self-employment (10.1%), a well-established career path. ANs, on the other hand, stood out for pursuing further studies (10.5%) to a much greater extent than in the three other groups (0.9% to 3.3%).

### Uncertain employment status (Study B)

Nursing occupations faced a more uncertain job future than MDs and OCs did, with more leaving their jobs. Surprisingly, more than one in ten ANs and RNs had not yet decided on their next career move, and 3.8% of ANs were transitioning into job seeking.

**Table 5:** Exit destinations for medical doctors (MDs), assistant nurses (ANs), registered nurses (RNs), and other licensed healthcare staff (OCs)

Exit destinations	MDs					ANs		RNs		OCs*	
	*n* = 676				%	*n* = 761	%	*n* = 2510	%	*n* = 1041	%
Self-employed	68		10,1^a^			18	2,4^b^	111	4,4^bc^	57	5,5^c^
Studies	6		0,9^a^			80	10,5^b^	84	3,3^c^	18	1,7^a^
Job seeking	11		1,6			29	3,8	36	1,4	23	2,2
Yet to be determined	63		9,3^ab^			86	11,3^b^	278	11,1^b^	62	6,0^a^
Other**	31		4,6^ab^			44	5,8^ac^	199	7,9^c^	21	2,0^d^
New employer:	497		73,5^ab^			504	66,2^a^	1804	71,8^b^	860	82,6^c^
Different Region	234		47,1^a^			63	12,5^b^	322	17,8^b^	147	17,1^b^
Municipality	10		2,0^a^			216	42,9^b^	693	38,4^b^	186	21,6^c^
State	28		5,6			26	5,2	98	5,4	50	5,8
Private	194		39,0^a^			168	33,3^b^	601	33,3^b^	433	50,3^c^
Other destinations	27		5,4			20	4,0	74	4,1	42	4,9
Missing	4		0,8			11	2,2	16	0,9	2	0,2

Note: Chi-square tests showed significant differences across occupational groups for all categories shown (all *p*<.001). Post-hoc pairwise comparisons with Bonferroni correction (α = 0.0083) were conducted. Within each row, different superscript letters (a, b, c, d) indicate significant differences between groups at *p*<.0083. Groups sharing the same letter do not differ significantly. For employment sector distribution, all pairwise comparisons were significant (*p*<.001) except Registered Nurses vs. Assistant Nurses (*p*=.064)

*Other licensed healthcare staff SSYK12 = 224 (Psychologist/Psychotherapist), 226 (Dentist), 227 (Occupational Therapist & Physiotherapist), 228 (Other healthcare specialists: Pharmacist, Audiologist, Dietitian, Speech Therapist, Optometrist), and 325 (Dental Hygienists)

** Other exit destinations: hourly employment, combination

## Discussion

This study aimed to provide a comprehensive understanding of staff mobility in the Swedish healthcare system. Adopting a two-part design that integrates a labour market mobility perspective with an organisational turnover lens, the analyses reveal not only how frequently staff leave their positions, but also where they go. By combining these complementary perspectives, the analyses move beyond traditional approaches that focus on single occupations or turnover as a single outcome. Instead, staff mobility is conceptualised as a dynamic process encompassing inflows, outflows, and internal movements within the healthcare system.

Our findings provide new insights into the dynamics of healthcare workforce turnover over the past decade. Although the insights focus on the Swedish healthcare sector, they are also relevant internationally. Over the last decade, turnover patterns in Sweden have shifted in ways that mirror global trends, including digital transformation, demographic ageing, and the impact of the Covid-19 pandemic. These patterns highlight systemic pressures and occupation-specific challenges, providing a more nuanced picture than typically portrayed in public discourse. In the following sections, we discuss the key findings from the labour market and organisational perspectives of the study and their implications for workforce sustainability and retention.

### Mobility patterns over time

Across the two parts of this study, a consistent picture emerges of relatively stable healthcare staff mobility over time. The stable proportion of employees leaving the public sector in Study A aligns with the stable patterns among MDs and RNs in Study B, indicating relatively steady healthcare staff mobility in the public sector from 2014 to 2024. This stability suggests that the overall turnover behaviour did not follow the shock-driven decision paths proposed by the *Unfolding Model of Voluntary Turnover* [37]. However, the two nursing occupations challenge the stability pattern. RNs had much higher mobility out of the private sector than the other groups, likely driven by increased outflows during the pandemic - a shock influencing mobility in line with the Unfolding Model of Voluntary Turnover [37]. In contrast, the rising external turnover among ANs in Study B is more consistent with *Job Embeddedness Theory* [38], which emphasises fit, social links, and perceived sacrifices in voluntary turnover. Given the shortest education, ANs probably make smaller sacrifices associated with a shift, a finding further corroborated by this group having the highest internal turnover level.

The value of the study design is illustrated by the apparent inconsistency between the two sub-studies. While study A shows that registered nurses (RNs) have low mobility out of employment in the public sector, study B reveals that RNs experience the highest level of external turnover among occupations. This can be explained by RNs typically not leaving public employment but rather transitioning between regional, municipal, and state-level employers, as indicated by Study B. Such mobility can help address regional workforce disparities, alleviate workforce shortages, and ensure that areas with fewer resources can still provide adequate care. For instance, when healthcare is reorganised, and hospital-delivered services are transferred to municipalities, it is advantageous for society that skills and competencies accompany these tasks, ensuring quality and efficiency across the overall system. At the same time, the outflow of OCs and MDs to private-sector employment identified across studies raises concerns about public-sector stability and the competence loss, underscoring systemic challenges in retaining personnel across sectoral boundaries. Even though most of these employees probably move to privately governed healthcare employers, some transitions can also be to private non-healthcare employers. Promoting sustainable career pathways and internal development may mitigate turnover and strengthen workforce sustainability in the public sector organisations. Future research should identify the causes behind and organisational practices that support retention and reduce sectoral outflows for specific occupations.

### Public and private sector dynamics

Our finding of significantly higher employment mobility out of the private rather than public sector challenges the narrative of a mass “exodus” of healthcare professionals from the public sector. While a larger share leaves private employment, the public sector’s larger size means more individuals leave it overall. Still, greater private-sector mobility aligns with previous research suggesting that healthcare professionals in the private sector are more likely to change employers, as public sector employment is generally more stable due to factors such as stronger job protection and more favourable retirement benefits [39]. Efforts to reduce reliance on agency staff in Sweden may also play a role [40]. This trend was strongest among RNs, with nearly one in five moving from private to public-sector roles in each two-year wave in Study (A) However, the private sector still constitutes a major destination for those changing employers, as shown in Study (B) This shows the importance of integrating complementary data sources to obtain a full picture of mobility patterns.

### A complementary organisational perspective

While aggregate patterns suggest relative stability, the organisational perspective reveals more dynamic processes at lower levels. External turnover consistently dominated each year during this period across all occupational groups (Study B), indicating that turnover primarily represents a net outflow rather than internal reallocation of staff. At the same time, staff members also transferred voluntarily to other divisions within the organisation. About 10% of employees left their divisions each year, with RNs showing the highest average external turnover (8%). However, both internal and external turnover may affect organisational performance, particularly during periods of labour shortages.

Comparisons with previous research show that turnover levels in the present study are moderate in an international context, although variation in measurement limits direct comparisons. In example, a Danish study reported 11% hospital staff exits in one year [41], and a Finnish study found that 13% of hospital employees actually left their positions by follow-up 2–4 years later [22]. A recent cohort-study from Sweden revealed that 40% of the midwives had changed position during a 3-year period [24]. Studies from other parts of the world have shown wide variation in nurses’ turnover rates. For example, in a study from South Korea, around 8% of nurses working shifts left their positions during a 12-month follow-up period [23]. In contrast, a review study reported far higher turnover rates in New Zealand (44%), Canada (20%), and Australia (15%) [42] Here turnover was defined as transfers or resignations of nursing staff from their primary employment positions, with variation in the degree of voluntariness and no information on the time spell. Across these studies, it is evident that the critique raised by Wynendale et al. regarding the lack of standardisation in measuring turnover in healthcare research [31] is highly relevant, even within the narrower research field of actual turnover. Nevertheless, in the light of these studies, the turnover among occupations in the present study appears less alarming than recent Swedish reports suggest [10, 15, 16].

### Implications for workforce stability

Given the ageing population, future workforce needs, and the low interest among younger generations in pursuing a healthcare career, avoidable turnover remains a pressing concern in healthcare. The goal for organisations is not to eliminate turnover but to maintain stable, low rates. While no universal threshold exists, turnover tends to be more costly in public labour markets [26], leading to generally lower optimal turnover rates. Within-division mobility often reflects organisational flexibility for addressing staff shortages, career progression, or restructuring.

However, voluntary turnover within divisions and organisations can have a particularly detrimental impact on organisational performance, especially if the most capable employees with better employment alternatives choose to leave. Costs vary by profession; replacing a physician is far more expensive than replacing staff with shorter education. Ultimately, turnover costs are particularly critical during labour shortages, as is currently the case in the Swedish labour market [8, 9]. Internal mobility, though preferable to external exits, still requires careful management at the division level, where disruptions affect service continuity and exacerbate the pressures on already strained healthcare systems. Drivers of internal mobility—such as dissatisfaction, high workloads, or limited opportunities for advancement—may mirror those behind external turnover, underscoring the need to address root causes for sustainable retention.

### Changing occupation or opting to an insecure future

Traditionally, turnover has been defined simply as an employee leaving a job [39, 43], but this overlooks the significance of where employees go next. As previous research suggests, turnover often involves a move to another work situation, and the factors driving such decisions, such as demographics or job conditions, may vary depending on the nature of the destination [37, 44]. Considering destinations allows organisations to better understand patterns and craft strategies for retention, development, and workforce sustainability [21, 45].

The findings from Study B revealed that most employees had secured new positions upon leaving their jobs, and Study A gave further insights into exit destinations. Transitions should ideally remain within healthcare to preserve skills and experience. In light of this, it is positive that MDs, a group with long education, often transition to self-employment or managerial roles within healthcare. In line with this, pursuing education is generally viewed as a positive career move, and with the shortest educational background, it is not surprising that ANs were the occupational group most often doing this. However, it is concerning that around one in three changing their occupation leave healthcare. This trend leads to a substantial loss of healthcare staff, further straining an already overburdened nursing workforce. In addition, more than one in ten ANs and RNs reported uncertainty about their employment status after leaving. This can be regarded as a signal to the healthcare system that something is amiss. Moreover, healthcare jobs often provide a sense of purpose and meaning, so leaving for an unpredictable future may lead to identity loss and heightened, compounding mental health challenges. Facing an uncertain future may indicate a life situation characterised by financial instability, outdated skills, and employment gaps that hinder re-entry. Together, these findings demonstrate that healthcare staff turnover is not a single phenomenon but comprises distinct and interrelated forms of mobility with different implications for workforce sustainability.

### Strengths and limitations

A key strength of this study is its two-part, multi-methods quantitative design, combining a labour market perspective (Study A) with an organisational perspective (Study B). By integrating three datasets based on actual turnover data, which allows for a thorough examination by integrating longitudinal analyses with in-depth investigations of organisational turnover, enhancing the validity and reliability of the study’s findings and conclusions. There is a risk of misclassification regarding whether a new SSYK code always indicates a transition to a different occupation rather than, for example, a change to a leadership position within the same occupation. Another limitation concerns the distinction between the “public” and “private” employment. Although respondents in both studies selected their response from predefined categories, these options depend on individuals’ interpretations of their employer’s organisational status rather than on the underlying funding structure. Further, the measure captures heterogeneous mobility types as it comprises mobility to other healthcare providers as well as to non-healthcare employers. This contextual complexity should be considered when assessing the implications of sector mobility for the capacity of the public healthcare workforce. The HR register data offers the advantages of complete coverage and the ability to distinguish among three types of workforce mobility, each with varying implications for organisations. However, organisations vary in retention policies, working conditions, geography, and other factors, which may limit the generalisability of the findings to a broader context. Additionally, we do not know to what extent respondents to an organisational exit survey, as in Study B, differ from non-respondents or how this might influence the results. Another limitation is the lack of data for non-response analyses, which could help clarify potential response bias in the two survey samples. The use of survey data introduces a risk of selection bias, as observed in the general SLOSH cohort, which overrepresents women, older individuals, married people, highly educated individuals, and those born in Sweden [36]. The large proportion of women across the five SLOSH sub-samples is mainly due to the highly female-dominated Swedish public sector but may still indicate overrepresentation. However, this problem is somewhat mitigated by the study design, which uses separate samples for each wave pair. Thus, gender bias is likely to have minimal impact. In addition, the SLOSH cohort may exhibit a healthy worker effect [36] as indicated by an overrepresentation of older healthcare workers, which may lead to underestimation of mobility. One final limitation of this study is that, although a significant and increasing proportion of healthcare workers are foreign-born, the data did not allow an investigation into whether mobility patterns vary by migration background. This aspect deserves further investigation in future research.

### Implications

The present study underscores the complex nature of mobility and turnover in healthcare, emphasising the need for a systemic and integrative approach to address these issues. By combining a labour market perspective (Study A) with an organisational perspective (Study B), and including multiple occupational groups, it moves beyond single-profession analyses and reveals substantial differences in turnover dynamics. Given healthcare’s collaborative nature, this multi-occupational perspective is essential. The findings highlight the necessity of retention strategies tailored to the unique mobility patterns of different professions and specific organisational contexts. Evidence from Denmark suggests nearly half of external turnover may be prevented through improvements in the psychosocial work environment, highlighting the significant potential of workplace interventions [41]. Leaving without a clear career forms part of broader instability, including sick leave and temporary staffing solutions [46–48]. To address complex turnover patterns, healthcare systems require a robust data infrastructure for ongoing, occupation-specific monitoring of mobility trends and causes - critical for evidence-based workforce management. Moreover, organisational instability compromises efforts to maintain a healthy and sustainable work environment [42, 49] and long-term workplace health and safety initiatives, particularly in contexts with high managerial turnover [50]. Thus, sustainable and supportive working conditions should be a strategic priority for healthcare organisations and policymakers seeking to enhance retention and ensure long-term system resilience. Additionally, coordinated efforts across sectors and institutions will be crucial to support career continuity and prevent avoidable turnover.

Few studies adopt the fundamental yet indispensable approach of systematically mapping trends before progressing to advanced analyses. As Burr notes, researchers often bypass these basic inspections of data, leaving our understanding of long-term developments fragmented and insufficient [30]. The two-part design of the present study responds to this gap by combining longitudinal trend analyses with detailed organisational data. Sweden’s healthcare system, with strong labour protections and rich administrative data, offers a valuable reference for understanding workforce resilience under global disruptions, and demonstrates how comprehensive data can capture turnover patterns more precisely.

## Conclusion

Labour shortages and high turnover in healthcare are major concerns, particularly in light of an ageing population. This study aimed to fill a knowledge gap regarding actual mobility and turnover patterns across Swedish healthcare occupations. By integrating findings from both the labour market and organisational components, the study reveals that the mobility has remained relatively stable from 2014 to 2024, despite the Covid-19 pandemic, but patterns differ by occupation and exit destination. Of particular concern are the substantial proportions of employees who depart without a clear destination, as well as those who move outside healthcare. These groups, specifically ANs, deserve focused attention in future research, as their trajectories may reveal deeper structural vulnerabilities in the healthcare labour market.

Future research should focus on understanding the factors that lead different segments of the healthcare workforce to distinct exit destinations, thereby enabling the development of tailored retention strategies.

## Data Availability

Study A: The SLOSH datasets generated and/or analysed during the current study are not publicly available due to restrictions from the ethical review board and considering that sensitive personal data are handled, but are available on reasonable request. Access to the data may be granted to other researchers in accordance with Swedish law, following consultation with the Stockholm University legal department. Requests for data, stored at the Stress Research Institute, Department of Psychology, should be sent to registrator@su.se with reference to ‘Healthcare staff mobility and turnover\_2026’ or directly to the corresponding author.Study B: The HR register data used in this study are available in the article. The exit survey data includes sensitive personal and organisational information that cannot be made freely available due to restrictions imposed by the ethical review board and the regional healthcare organisation. Access to the data may be granted to other researchers after consultation with the regional organisation and subject to additional ethical approval. All requests for data access should be directed to the corresponding author.

## Footnotes

1 In contrast to doctors, nurses, and the group of other licensed health professions, assistant nurses in Sweden are not a licensed profession but have held a protected professional title since 2023, restricting the use of the title to individuals who meet nationally defined competency requirements.

2 In Sweden, regions are responsible for organising, financing, and providing healthcare services [28, 29]. The region is organised into several operational divisions (Swedish: ‘förvaltningar’), which constitute the main organisational units for healthcare delivery. Most divisions correspond to hospital-based organisations, while some provide region-wide support services.

## Declarations

### Use of AI

During the preparation of this work, the authors used Grammarly for language editing. After using this tool, the authors reviewed and edited the content as needed and take full responsibility for the publication’s content.

## Abbreviations

AN: Assistant Nurse
GEEs: Generalized estimating equations
HR: Human Resources
MD: Medical doctor
OC: Group of other licensed healthcare occupations
RN: Registered nurse
SLOSH: The Swedish Longitudinal Occupational Survey of Health
SSYK: The Swedish Standard Classification of Occupations
SWES: The Swedish Work Environment Survey
